# A Log-Level Data-Driven Precision Education Tool for Pediatrics Trainees: Human-Centered Development and Validation Study

**DOI:** 10.2196/79952

**Published:** 2026-02-23

**Authors:** Alexander Fidel, Mark V Mai, Naveen Muthu, Adam C Dziorny

**Affiliations:** 1 Center for Healthcare Quality and Analytics, Children's Hospital of Philadelphia, 734 Schuylkill Ave, Philadelphia, PA, 19146, United States, 1 602-284-8098; 2 Department of Pediatrics, School of Medicine, Emory University, Atlanta, GA, United States; 3 Department of Pediatrics, School of Medicine, University of Rochester, Rochester, NY, United States

**Keywords:** planned learning, human-centered design, HCD, decision support, experience tracking, human-computer interaction, cognitive ergonomics, human factors engineering

## Abstract

**Background:**

Exposure to patients and clinical diagnoses drives learning in graduate medical education (GME). Measuring practice data, how each trainee experiences that exposure, is critical to planned learning processes including the assessment of trainee needs. We previously developed and validated an automated system to accurately identify resident provider-patient interactions.

**Objective:**

In this follow-up study, we use human-centered design methods to meet two objectives: (1) to understand trainees’ planned learning needs and (2) to design, build, and validate the usability and use of a tool based on our automated resident provider-patient interaction system to meet these needs.

**Methods:**

We collected data from 2 institutions new to the American Medical Association’s “Advancing Change” initiative, using a mixed methods approach with purposive sampling. First, interviews and formative prototype testing yielded qualitative data that we analyzed in several coding cycles. We built interview guides to collect data required for a work domain assessment, learning use case elicitation, and ultimately design requirement identification. We structured coding efforts within 2 existing theoretical models. Feature prioritization matrix analysis then transformed qualitative analysis outputs into actionable prototype elements that were refined through formative usability methods. Finally, qualitative data from a summative usability test validated the final prototype with measures of usefulness, usability, and intent to use. We used quantitative methods (eg, time on task and task completion rate in summative testing).

**Results:**

We represented the GME work domain assessment through process-map-design artifacts that provide target opportunities for intervention. Of the identified decision-making opportunities, trainee-mentor meetings stood out as optimal for delivering reliable practice-area information. We designed a “midpoint” report for the use case of such meetings. We arrived at a final prototype through formative testing and design iteration. This final version showed 5 essential visualizations. Summative usability testing resulted in high performance in subjective and objective metrics. Insufficient baseline data were captured to draw comparative conclusions in a formal evaluation against existing tools or workarounds to support planned learning. However, the prevailing reported absence of tools and the ad hoc nature of approaches that do exist strongly imply an unmet need for the type of usable summary method delivered in our tool. We collected data from June 2021 through September 2023. Eight resident physicians composed the validation sample, including 4 (50%) residents from the Children’s Hospital of Philadelphia and 4 (50%) residents from the University of Rochester Medical Center.

**Conclusions:**

We describe the multisite development of a tool providing visualizations of log-level electronic health record data, using human-centered design methods. Delivered at an identified point in GME, the tool is ideal for fostering the development of master adaptive learners. The resulting prototype is validated with high performance on a summative usability test. Additionally, the design, development, and assessment process may be applied to other tools and topics within clinical informatics.

## Introduction

Experiential learning through direct patient care is an important paradigm in graduate medical education (GME) [1]. Readily available measures of patient exposure are necessary to quantify gaps in trainee experience [2]. Such objective tools can provide data to guide a trainee’s individualized education plan and enhance future learning [3,4]. The reflective practice and precision education (PE) conceptual models build on the Master Adaptive Learner (MAL) model, pointing to patient exposure data powerfully informing the planning phases of trainee education [5-7]. Planned learning in this context amounts to educational experiences that are structured by the institution, but also extends to intentional, goal-directed behavior arising organically from metacognition surrounding practice. Planned learning is integrated into the residency or fellowship curriculum either by program directors or the learners themselves. It can be the decision to take one elective over another, but it can also be the decision to look out for and seize opportunities to get exposure to a certain patient population or procedure. It is distinct from informal, spontaneous learning that might occur during daily patient care without specific predefined objectives or evaluation methods. This intentionality is anchored in the MAL model, which holds that material learning occurs by cycling through states of planning, learning, assessing, and adjusting.

Using practice data, trainees may situate their experience within their cohorts, identifying targeted opportunities for learning [6]. Although manually tracked case logs by trainees themselves show promise in providing such data, those methods are labor-intensive and limited in their ability to scale [8,9]. Several technology-based systems have been developed to identify trainees’ patient experiences automatically and accurately across rotations [10-12]. PE of this kind in GME has recently been advanced as a data-driven approach for improving personalization and efficiency of learning, assessment, and feedback by using longitudinal learner data and analytics. In this framing, programs move beyond aggregate case logs or episodic evaluations toward fruitful cycles of the MAL [13,14]. And while reliable characterization of each trainee’s clinical experiences at deep granularity has emerged as a key PE enabler, it remains unknown how to optimally deliver this information to trainees [15,16].

GME trainees need targeted delivery of specific information and knowledge to enhance their educational experience. *Educational* decision support (EDS) deals with such information [17]. We posit that EDS systems are as necessary as clinical decision support (CDS) systems that support clinical duties. To this end, authors initially developed and validated TRAILS (Trainee Individualized Learning System), an automated software platform that uses electronic health record (EHR) metadata—particularly audit logs, note authorship, order placement, and care team assignments—to identify clinically meaningful resident provider-patient interactions across multiple care settings for medical education tracking and assessment [11,18]. We used EHR audit log time stamps and algorithmically calculated interaction intervals by summing time gaps between consecutive events to derive total time-in-chart, a computed metric with strong predictive power to add to raw metadata features. Data cleaning steps like exclusion of data when total time-in-chart is less than 1 hour ensured the models accurately distinguished genuine clinical interactions from incidental chart access. We then applied logistic regression classifiers trained on resident-validated resident provider-patient interactions.

To be useful and usable, EDS output like that of TRAILS must deliver the right information to the right subset of users, at the right time, through the right information channels, and in the most usable format [19]. Traditional technology-centered design (TCD) approaches are insufficient, with outcomes of increased complexity for users, elevated error rates, poor intention to use, and abandoned adoption [20,21]. TCD approaches fit an interface to work-as-imagined and engage users late in the process, if at all. The alternative, human-centered design (HCD), begins by assessing user goals, tasks, abilities, and cognition prior to design ideation. By first identifying contextual needs through requirements elicitation with end users, followed by iterative prototyping and formative evaluation, HCD interface creation instead fits work-as-done, and better results follow: simplified use, low error rates, high affinity, and eventually, high adoption [22,23].

The objective of this study was to follow an HCD approach to design and develop a user interface overlaying an existing automated EDS system, and to measure the summative usability of the design product. Specifically, we sought to: assess the trainee learning work domain and overall context for system implementation; use this knowledge to inform initial designs; apply formative and summative evaluation techniques to the tool; use mixed methods for data collection consistent with HCD standards. We hypothesized that this approach would generate a prototype, meeting acceptable benchmarks for technological acceptance, predicting eventual adoption. The endorsement of such a prototype, assessed to have good usability, would support that our approach could be generalized to other medical applications. The HCD approach we targeted was synthesized across diverse health innovations and health IT including CDS [24-26]. It is also supported by systematic reviews in broader human-computer interaction or software-engineering contexts [27,28]. These suggest that any one viable HCD method is transferable beyond a single specialty or use case.

## Methods

### Study Design

We performed a prospective mixed methods study within 3 distinct phases of data collection (Table 1) and analysis. Participants were physicians from 2 large academic institutions: the Children’s Hospital of Philadelphia (CHOP) and the University of Rochester Medical Center (URMC). Annual trainee cohort sizes are over 500 and over 900, respectively [29,30]. These programs cultivate cultures of planned learning by embedding residents in mentored research projects and data-driven quality improvement teams where they critically reflect on performance metrics, identify specific gaps in outcomes, and develop targeted interventions that close those gaps through iterative, supervised cycles of implementation and assessment [31,32]. We conducted the study over a 2-year period, from June 2021 to September 2023.

**Table 1.** Data collection methods.

Principal data collection method	Participant recruitment^a^	Moderation^b^ and setup	Data
Semistructured interview	Trainees and individuals responsible for trainee coaching and program-level planned learning.	Participant was asked questions from role-appropriate interview guide	Recorded and transcribed session Moderator notes
Prototype walkthrough interview	Trainees and faculty mentors	Participant was given most recent prototype iteration of planned learning tool and asked to share screen during walkthrough	Recorded and transcribed session Moderator notes
Summative usability test	Residents	Participant was given final prototype of planned learning support tool, the “MV^c^ midpoint report” and directed to complete test on REDCap^d^ (Vanderbilt University) using the report	Time-stamped, REDCap-collected answers to task questions Responses to TAM^e^-based Likert psychometrics assessing PU^f^, PEOU^g^, and scenario realism.

a All samples purposive; interview phase saturation was reached succeeding first session when no new data could be synthesized in analysis; repeat participation in or across phases was disallowed.

b Each session in all methods was conducted with 1 participant at a time with 1 moderator over Teams (Microsoft) or Zoom (Zoom Communications, Inc) over 1 hour.

c MV: minimum viable.

d REDCap: Research Electronic Data Capture.

e TAM: technology acceptance model.

f PU: perceived usability.

g PEUO: perceived ease of use.

For coding paradigms in all qualitative research, a 4-member coding team reached cooperative consensus. All had full access to the complete qualitative datasets. Each team member independently reviewed first-cycle coding of all raw data prior to weekly consensus sessions. In these meetings, the team interrogated discrepant interpretations and pursued dialogical intersubjectivity until group consensus was reached. We also refined codebooks in this way, revising, collapsing, or generating new codes, categories, and themes as warranted.

### Work Domain Assessment

The first phase of an HCD process, work domain assessment, identifies individuals’ roles and details the overall context or work system in which a prototype tool would be used [33,34]. We conducted semistructured interviews with a purposive sample of stakeholders involved in trainee-planned learning. Collecting data from all roles involved is essential to characterize a sociotechnical system of clinical work accurately [35]. Role-specific interview guides covered 2 subjects: trainee sensemaking and decision-making in practice-area learning; mentor or program perspectives on entrustment ladders and how practice-area gaps are identified, discussed, and closed (Multimedia Appendix 1).

We coded the corpus of qualitative data (Table 2). Notably, we borrowed constructs in the Systems Engineering Initiative for Patient Safety to create early cycle topic codes [35,36]. From early cycle coding analysis, we created process maps visually representing dynamics of planned learning.

**Table 2.** Qualitative analysis methods used in the study.

Coding type	Purpose	Output
Descriptive coding [37]	Identify topics in the interview corpus.	Table of quotes from interviews and their labels
Provisional coding [36]	Deductively organize topics into categories using a “start list” code book from SEIPS^a^ [35]. Improve the design team’s grasp of users [38,39].	Expanded table with labels fit to topics and categories
Process coding and causation coding [40,41]	Abstract dynamism or change in topics or categories into graphical representations: SEIPS topics acting as barriers or facilitators to learning; mental models acting on decisions; evolving intentions, choices, objectives, values, perspectives, needs, desires, and agency.	Process maps explaining contexts, conditions, interactions, and consequences of planned learning
Inductive coding [42]	Abstract process map and code table data into questions our users ask, answerable with trainee EHR^b^ metadata filtered on clinically meaningful rPPIs^c^ and encounter context fields required by that question (eg, care setting or elective label).	Column in feature prioritization matrix
Magnitude coding [43]	Assigns each user question an impact score: numerical value (from 1 to 5 where 5=“high impact”) showing impact an informed answer would have to planned learning weighing use of the described learnings and degree of relevance to scenarios described in interviews.	Column in feature prioritization matrix

a SEIPS: Systems Engineering Initiative for Patient Safety.

b EHR: electronic health record.

c rPPI: resident provider patient interaction.

### Formative Usability Test

**Figure 1.**
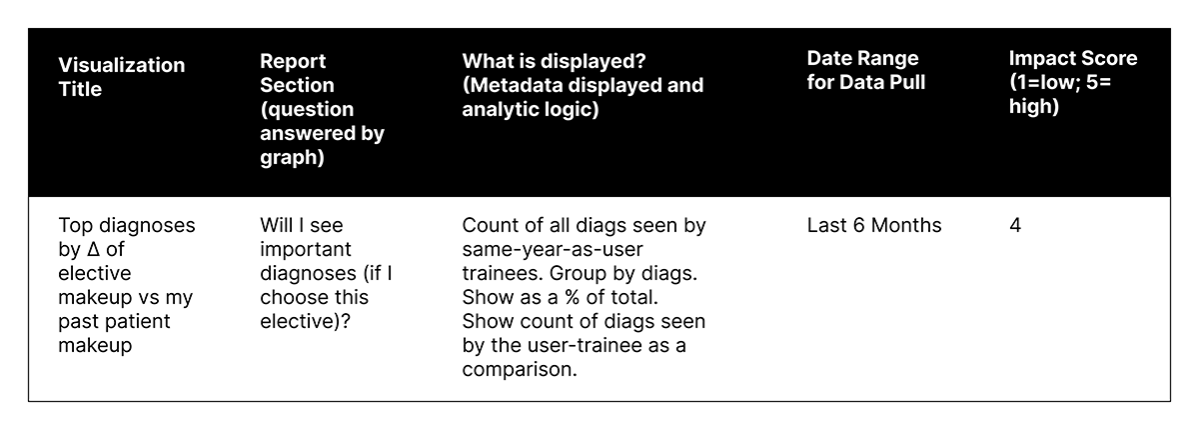
An example of a single record from the feature prioritization matrix.

To begin formative testing, we required an initial prototype. We initiated further coding processes (Table 1), first inferring key user questions, such as “Will I see important diagnoses if I choose elective X?” These questions formed the basis of an important design artifact in HCD, the feature prioritization matrix (FPM; Figure 1).

**Figure 2.**
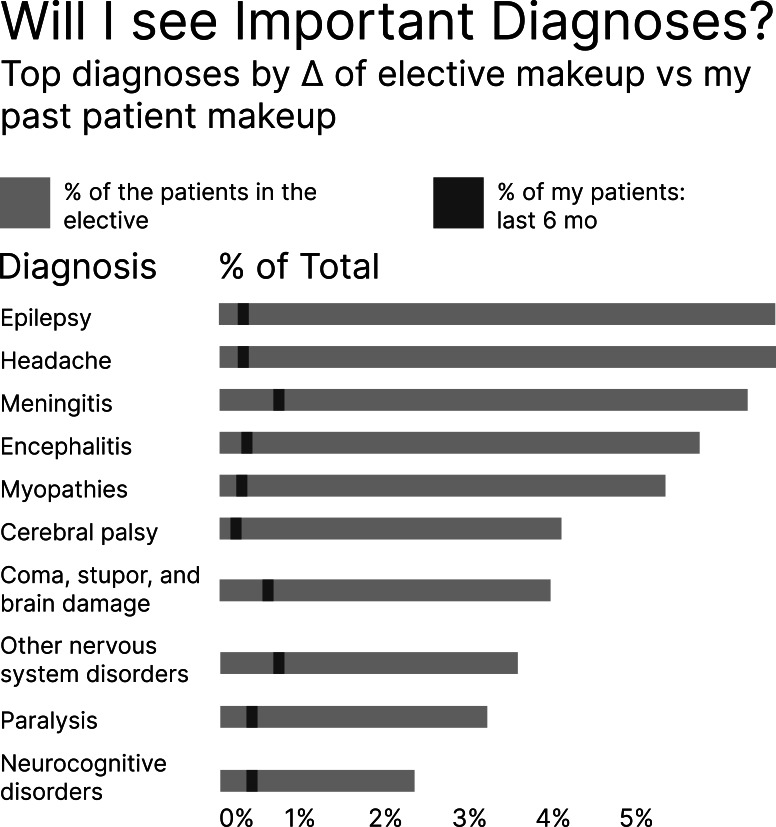
The corresponding visualization in the report from the feature described in Figure 1.

We produced an initial prototype of a planned learning support tool using the FPM from a collaborative unmoderated design session informed by reviewing recently completed process maps. We reached consensus on data visualizations that would allow trainees to identify or close gaps in experience (Figure 2). This action is the core of planned learning, and we formed the first report with visualizations supporting it.

We then iterated on the design in interview walkthroughs with users. With each walkthrough, newly elicited features and corresponding visualizations were added to the FPM and report. Using an impact metric from the completed FPM (Multimedia Appendix 2) and descriptions of required data for each visualization, we determined which features the current system architecture could feasibly support. We then produced a final version of our planned learning tool, the “minimum viable (MV) midpoint report,” including only the most impactful, feasible visualizations (Multimedia Appendix 3).

### Summative Usability Test

**Figure 3.**
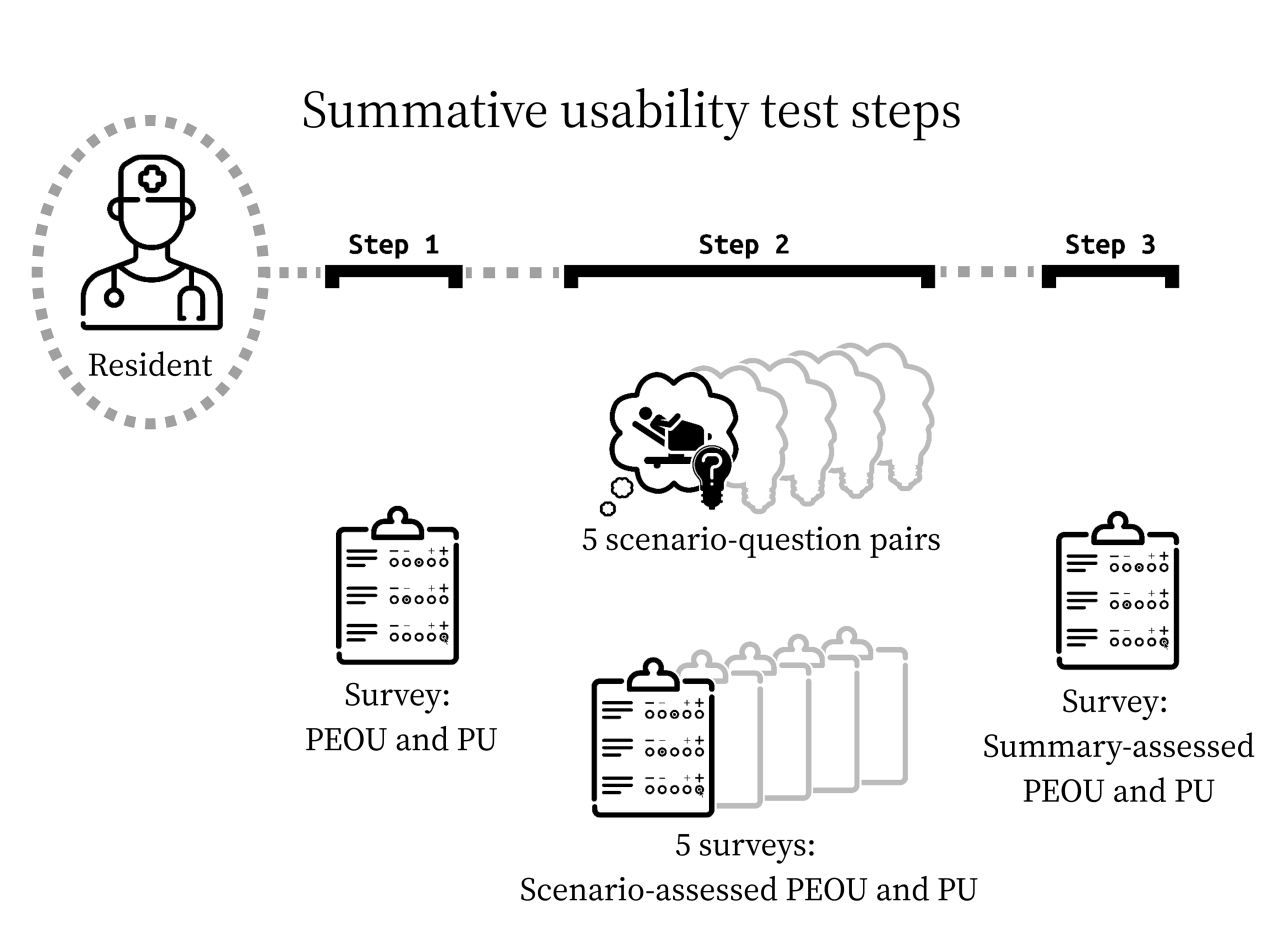
Summative usability test procedure for a single session, where a resident participant moves through the 3 steps, guided by a moderator. PEOU: perceived ease of use; PU: perceived usability.

Whereas formative usability testing is meant to expose design flaws and drive prototype iteration forward, summative usability testing is meant to validate a prototype that designers theorize is ready for deployment. We developed a summative usability test to assess usability and use of the “MV midpoint report” and measure performance on MAL planning-related tasks using the report (Multimedia Appendix 4). Participants completed 5 tasks (Figure 3). For each task, participants used the report to answer one of the following key user questions:

1. What are the diagnoses to which I have received the most exposure?
2. In what clinical environments am I seeing them?
3. What gaps in diagnosis exposure could I be filling?
4. What is my exposure to acuity and complex care?
5. In each elective available to me, will I see important diagnoses?

In this way, the final cycle of analysis and design in the HCD process would be complete (Multimedia Appendix 5).

For each task, we developed a scenario with realistic clinical data and a single complementary test question to which an MV report feature offered an answer. The purpose of each scenario-question pair was to assess usability and use. Four of the 5 scenarios were paired with a multiple-choice question. A fifth scenario was paired with a free-text response. Participants in this usability test were thus assessed with these 5 scenario-question pairs.

Participants were given the MV report in advance of the test for optional review. Participants were told to suspend disbelief and answer questions in the test as though the report were their own, aggregating their own practice area data and encountering the described scenarios during residency.

Participants completed a pretest questionnaire that asked postgraduate year of the participant, whether and how they kept records of their practice areas, and if so, perceived usability (PU) and perceived ease of use (PEOU) of that system. This quantified the usability of participants’ baseline approaches to tracking practice-area exposure (if any) for comparison against our tool. PU and PEOU questions were adapted from the development of another successfully implemented CDS tool (Table 3) [44].

**Table 3.** Measures within the summative test and the group of user beliefs which they assess.

Measure^a^	Score group
This scenario is important in finding learning or entrustment gaps and resolving them.	Test realism
This scenario is realistic in the search for learning or entrustment gaps and resolving them.	Test realism
In this scenario, the system was easy to use.	PEOU^b^
In this scenario, the system allowed me to perform tasks efficiently.	PEOU
Overall, I am satisfied with how the system is designed in this scenario.	PEOU
In this scenario, the system provided useful features.	PU^c^
In this scenario, the system provided useful information.	PU
In this scenario, the system is an improvement over what I would have used before.	PU

a Measures were assessed on a Likert scale from 1 (strongly disagree) to 9 (strongly agree). Participants were assessed after each scenario and then once more in a summary assessment at the end of the test.

b PEUO: perceived ease of use.

c PU: perceived usability.

We report these descriptive statistics for the groups of measures that assessed PU and PEOU immediately after scenarios, PU and PEOU summarily at the very end of the test, and for the group assessing the realism of the scenarios. Scenario-question pairs were scored for correctness and used to calculate the task completion rate (TCR) by dividing correct answers by total answered questions. We also report average task completion time as the amount of time spent from a scenario-question load page to the submission of a *correct* response.

### Ethical Considerations

This study protocol was reviewed by the institutional review boards (IRBs) of both CHOP (IRB 18-014866) and URMC (study 00006717) and determined to meet exemption criteria. We obtained a waiver of written informed consent from both IRBs. Participant data were deidentified and stored by random identifier to protect subject privacy. The participants were not compensated for their participation.

## Results

### Work Domain Assessment Results

We interviewed 8 participants (Table 4). We deployed initial descriptive and provisional coding efforts. Starting from the top of the codebook, 4 People, Environment, Tools, and Tasks scan categories were decomposed into 34 topics which yet further broke down into 57 subtopics. Multimedia Appendix 5 links key quotations from the interviews for each of the People, Environment, Tools, and Tasks categories and traces their impact forward through all study phases.

**Table 4.** Participants for all study phases.

Phase	Purpose	Sample
Work domain assessment (interview)	Understand work system or planned learning decisions	2 trainees, 5 program directors, and 1 administrator
Formative usability testing (prototype walkthrough)	Iterate report design	6 mentors and 1 resident
Summative usability testing	Validate EDS^a^ tool (MV^b^ midpoint report) with end users	8 residents

a EDS: educational decision support.

b MV: minimum viable.

**Figure 4.**
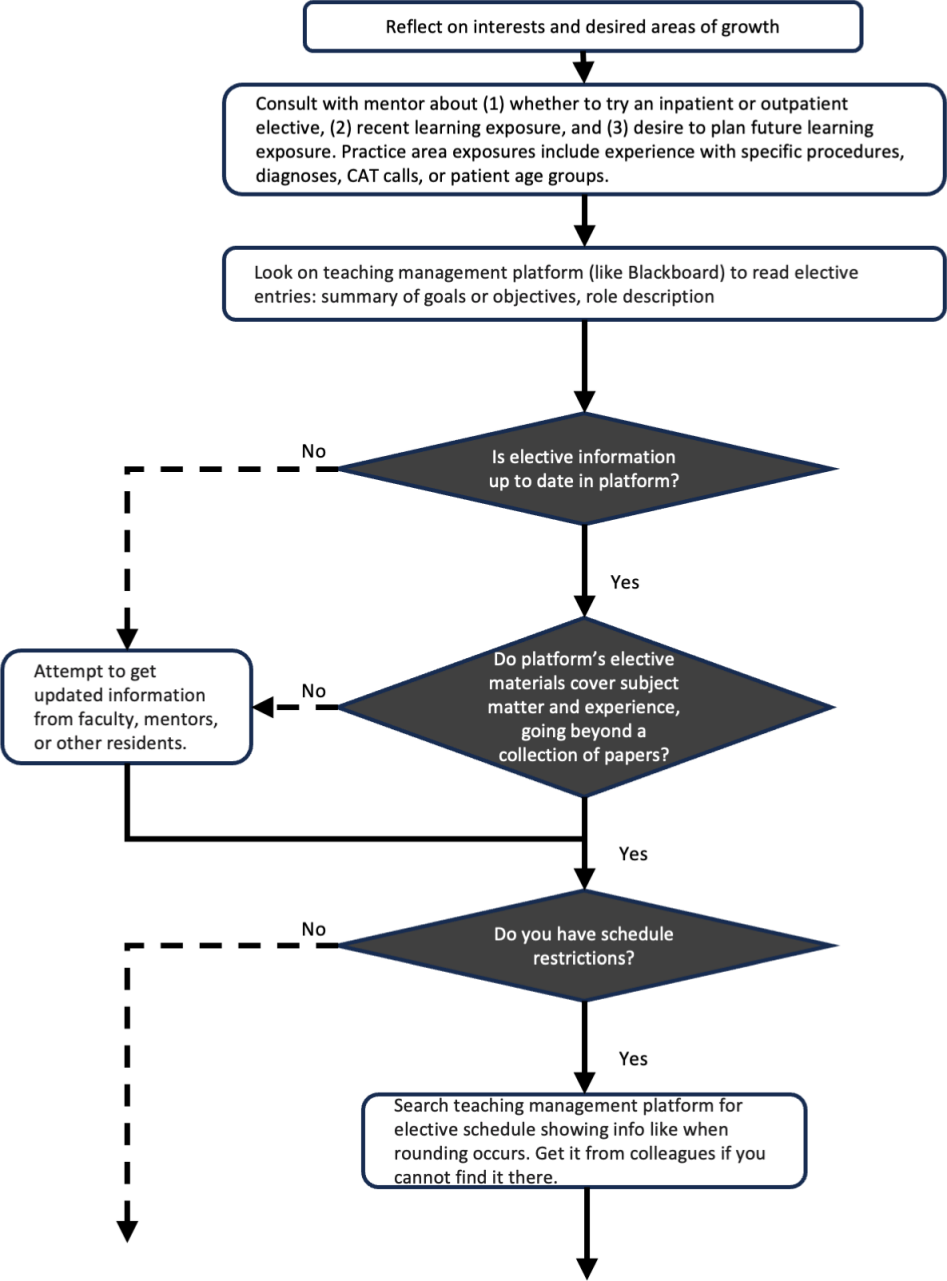
A segment of 1 of the 7 process maps. Diamonds denote decisions and rectangles stand for events, actions, or changes in mental model. CAT: Critical Activation Team.

Through our process or causation coding and process map analysis, we produced 7 maps describing processes across 3 roles: trainees (Figure 4), trainee mentors or program directors, and program administrators. The documents mapped 52 decisions or forks in the processes with 128 other steps, actions, or changes in the mental model (Multimedia Appendix 6).

We first established that mentors and program directors had fewer existing assessment workflows than trainees. While their leadership and expertise amplified potential impact they could achieve with better practice data, trainees had a far greater volume of opportunity to identify gaps and close them for themselves. Residents, in particular, had the highest number of planning and assessment opportunities. While other populations could still be influential secondary users of an EDS, residents emerged as optimal primary users of a decision support tool targeting planned learning.

Reviewing the process maps, we determined that clinic and rotations were high-inertia settings with high mental stimulation for our trainee users. The heavy demands on cognitive resources meant that planning in these situations would be secondary to a primary use case that also emerged from the data.

**Figure 5.**
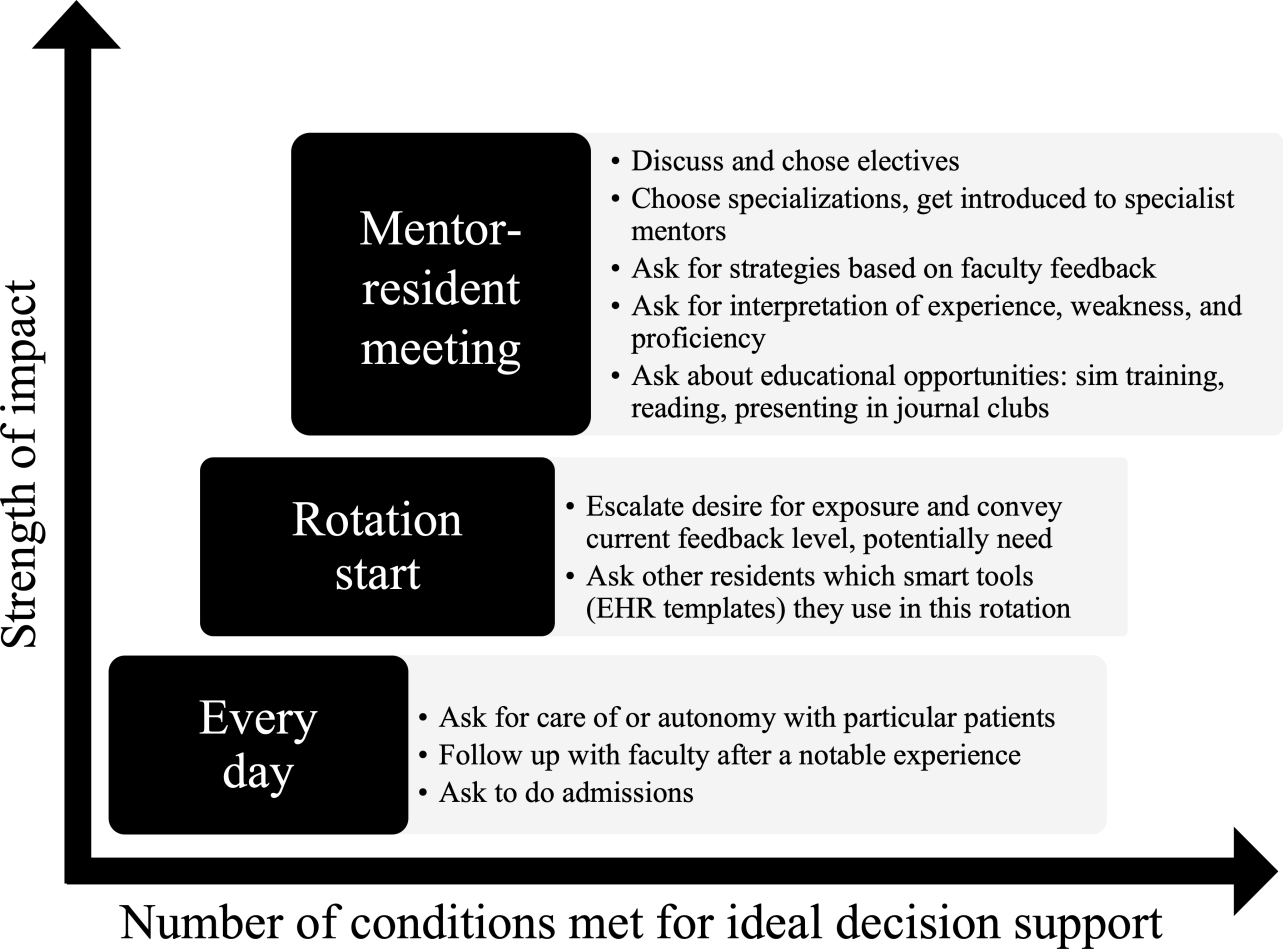
Settings where a report of practice area data could be leveraged and planned learning opportunities for each. These are plotted against qualities the research team used to select the target setting of the report. EHR: electronic health record.

Alternatively, trainee-mentor meetings, conducted much less frequently, stood out as a key target experience. By the time trainees meet with their mentors, they have usually formed some idea of a learning state gap but lack the expert system knowledge required for planning its closure. The MAL model holds that planned learning occurs by cycling through states of planning, learning, assessing, and adjusting. These meetings offered a calm moment when trainees and mentors cooperatively identify gaps, make plans to close them, or assess those efforts. This is the critical work of the planning and assessing phases. This trainee-mentor meeting environment had other benefits. It also allowed for contingency plans and provided a structure for replanning in future meetings, both of which are optimal planning practices from the standpoint of human factors engineering and cognitive science (Figure 5).

### Formative Usability Test Results

**Figure 6.**
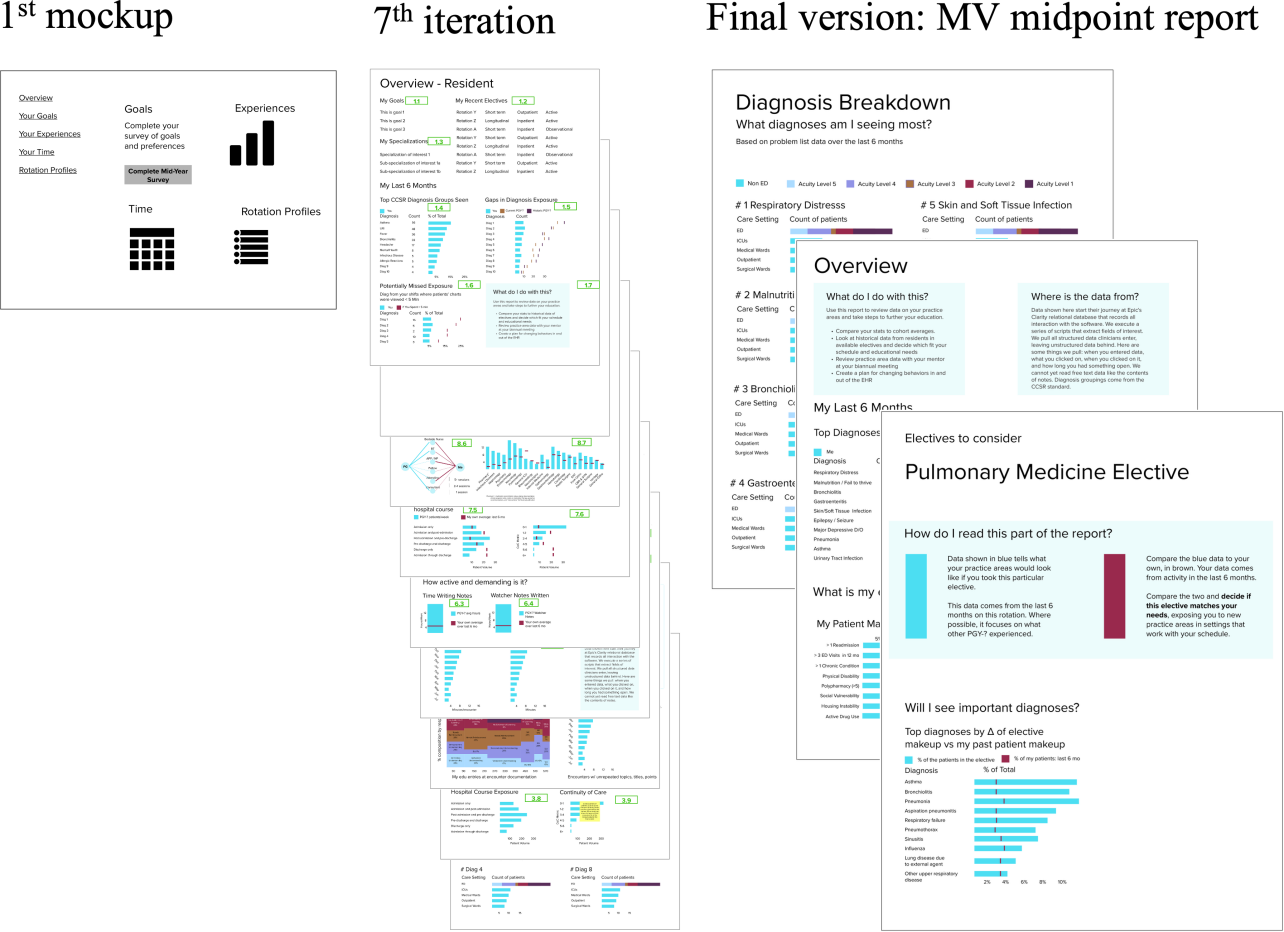
Progress of the initial report to the penultimate iteration to the final minimum viable (MV) midpoint report used in summative usability test.

Based on the results of our workflow assessment, we designed an initial report containing a single page of placeholder visualizations. Seven participants (6 program directors and 1 resident) completed the formative usability test, iterating from this initial version. Iterations to the midpoint report were made following each test, adding new and altering extant features. The seventh version of the report following the final formative interview contained 51 distinct visualizations of trainee past and potential future data, spanning nine pages (Figure 6). The complete FPM contained a corresponding 51 records (Multimedia Appendix 6). Following feature reduction, the MV midpoint report contained 5 visualizations over 3 pages, assuming one elective under consideration where an additional page would be added for each elective.

### Summative Usability Test Results

Eight resident physician participants in total completed the summative test; 4 each from CHOP and the URMC (n=4, 50%). The TCR across all 5 scenario-question pairs, defined by the selection of the correct answer to a scenario’s accompanying multiple-choice question, was 78%. Mean task completion time was 2 minutes and 39 seconds (SD 2 minutes and 30 seconds). Results broken out by each of the 5 scenario-question pair tasks (Table 5) show that completion-time variance was primarily driven by task 1 which showed a much longer mean time and wider range than the other tasks. This may have included orientation time with the structure of the test as well as the MV midpoint report. Tasks 3 and 4 most negatively impacted overall TCR. This may be attributed to more salient, earlier visualizations in the report being used in favor of more setting-specific data breakdowns required for these questions. The summative usability test data and capture plan did not allow for more thorough error type analysis.

**Table 5.** Summative test results by task (N=8).

Task	Completion time (min:s), mean (SD)	Task completion rate (%)
1	04:31 (4:18)	88
2	01:40 (0:36)	88
3	03:28 (1:24)	63
4	02:43 (1:00)	50
5	01:19 (0:48)	100

The pretest questionnaire provided baseline findings. Seven of the 8 participants reported no systematic method for tracking their practice-area exposure. Only 1 participant reported using an EHR-based workaround (described in a free text survey field as “saving patient reports on Epic”), and rated this approach 5/9 for both PU and PEOU (scores 6‐9 considered positive).

PU and PEOU scores for the MV midpoint report were high (Table 6). The median scores for scenario-assessed PU and scenario-assessed PEOU were 8 (IQR 5-9) and 8 (IQR 6-9), respectively. Scenario realism received a median score of 8 (IQR 6-9). Distinct from the scenario-assessed PU and PEOU that followed each of the 5 scenarios, a single summary questionnaire assessed the same measures at the end of the test. The median summaries for PU and PEOU were 8 (IQR 8-9) and 7.5 (IQR 7-8.25), respectively.

**Table 6.** Performance of the education decision support tool in terms of usability and scenarios in terms of task fidelity in summative usability testing (N=8).

Score group	Median^a^ (IQR)
Scenario-assessed (per-task) measures	
Perceived usefulness	8 (5-9)
Perceived ease of use	8 (6-9)
Summary (holistic post-test) measures	
Perceived usefulness	8 (8-9)
Perceived ease of use	7.5 (7-8.25)
Test realism	8 (6-9)

a Scale from 1 (nonperformant) to 9 (performant).

## Discussion

### Principal Findings

In this study, we describe the HCD process used to develop and assess a user interface to an EDS system that we find likely to be adopted. Like many designs for complex sociotechnical systems, an initial phase to better understand the implementation context led to substantial changes in our design goal. We shifted from a tool that trainees might use regularly to a tool for longer-term planning in the context of trainee-mentor meetings and potential choice of future elective rotations. We subsequently followed a typical iterative design and formative testing process. Upon achieving a stable “MV” design, we conducted our summative testing. Given that our goal in summative testing is to predict future adoption of the tool by trainees, we supplement typical usability measures of task completion, efficiency, and perceived satisfaction with an assessment of perceived usefulness and intent to use, consistent with the technology acceptance model (TAM) [45].

Systems have been judged above average in their usability when TAM-based questionnaires reach 80% [46]. High TAM usability scores signify intent to use a tool, and intent is the highest predictor of adoption [45]. This predictive factor is strengthened by high TCR [47]. Unfortunately, it is known in usability research that meta-analysis has not set completion rate benchmarks [48]. Deference, however, to TCR of similar tools at testing is standard practice in their place [49]. In comparable analytics software from education or clinical data analysis in sensemaking, reflective contexts (as opposed to CDS for real-time use preceding immediate, nonreversible patient action), we find similar TCR [46,50,51]. Matching this standard, we may draw the conclusion that our scores suggest the likely adoption of the tool.

The Accreditation Council for Graduate Medical Education requires trainees to experience a range of diagnoses during graduate medical training [1]. Using an EDS system, we can facilitate appropriate diversity of patient experiences and better quantify outcomes in GME using informatics tools [52-54]. Previous efforts to attribute trainee patient experiences have been limited in scope and dissemination. Several automated systems rely solely on EHR data to link clinical practice directly with education to personalized, timely outcomes [10,16,53]. However, these systems do not attribute patient experiences across clinical contexts or institutions. Dashboards to facilitate the feedback of clinical data to both GME and to continuous professional development have had mixed effectiveness, citing development without proper learning system stakeholder involvement and the absence of facilitated precepting to understand their learning gaps [55,56]. Our tool follows established HCD methods to include those users in interaction conception and make precepting intuitive.

With the usability and PU demonstrated in the summative test, we will implement the MV report to assist trainees with a number of tasks: visualizations of their top diagnosis types seen; their gaps in exposure compared to their peers and historical comparisons; their exposure to complex care patients; and most importantly, diagnosis makeup of electives or clinics that trainees could choose to further their exposure in weak areas of knowledge. The report will be delivered to trainees and their mentors before scheduled meetings biannually and will be discussed by the pair. Among other decisions that the report may inform, it will simplify the critical choice of electives or clinics that trainees can choose to better close their training gaps.

### Limitations

The formative testing sample over-represented mentors or program leaders relative to trainees; however, the final MV report was evaluated with resident end users in summative usability testing.

The study is limited by the sample size at 2 institutions, which may not represent the breadth of GME experience. Because of this, projected adoption based on high scores on PU and PEOU assessments may not be generalizable, and ongoing measurement will be necessary during implementation.

The level of realism achieved in the simulations required participants to suspend disbelief. All participants had experience and context to create clear mental settings from experience in the medical education system, though the fidelity achieved by descriptive text in immersing participants in that reality is low. In addition, the level of realism was tempered by technological limitations. We could not use actual trainee data from each specific participant or generalized trainee data from each site. Reports were populated by data that did not map to participants’ clinical experience, instead showing a generalized portrait of an “average” resident validated by clinical experts, requiring further suspension of disbelief.

Future work will improve validity deficiencies by using trainee-specific data in usability testing in a multicenter study with a greater sample drawn from more institutions, additionally including error type analysis of failed tasks. Exploration should also attempt to draw insights from beyond the EHR to triangulate them from multiple datasets including those from sources like training management systems, health care workforce management platforms, and other self-reported data. Similar efforts are already underway to support professional development [57,58]. A follow-up longitudinal study should examine click-stream engagement metrics, long-term post-deployment satisfaction via psychometrics, and downstream effects, such as changes in elective selection patterns, increased exposure in previously underrepresented diagnoses, and improvements in competency-based assessments.

### Conclusions

We iteratively developed and performed usability testing on a 5-visualization report displaying trainees’ aggregate practice area data from the EHR. Our results indicate a high likelihood of the report’s adoption as an effective tool in graduate medical education, aligning with the MAL planning phase tasks and allowing them to be completed in a timely manner. We assessed use and usability with instruments and results that predict significant future adoption. Evolving research will examine downstream effects on entrustment following implementation. This study provides a successful user interface for functions executed from an educational decision support system and offers a foundation for further research of clinical and educational applications of this system.

## Supplementary material

10.2196/79952 Multimedia Appendix 1 Work domain assessment interview guides.

10.2196/79952 Multimedia Appendix 2 Feature prioritization matrix.

10.2196/79952 Multimedia Appendix 3 Final prototype minimum viable (MV) midpoint report.

10.2196/79952 Multimedia Appendix 4 Summative usability test.

10.2196/79952 Multimedia Appendix 5 Quotes and coding leading to summative tasks.

10.2196/79952 Multimedia Appendix 6 Process maps.

## Abbreviations

CDS: clinical decision support
CHOP: Children’s Hospital of Philadelphia
EDS: educational decision support
EHR: electronic health record
FPM: feature prioritization matrix
GME: graduate medical education
HCD: human-centered design
IRB: institutional review board
MAL: Master Adaptive Learner
MV: minimum viable
PE: precision education
PEOU: perceived ease of use
PU: perceived usability
rPPI: resident provider patient interaction
TAM: technology acceptance model
TCR: task completion rate
TRAILS: Trainee Individualized Learning System
URMC: University of Rochester Medical Center
