# Supplementary material for: A Log-Level Data-Driven Precision Education Tool for Pediatrics Trainees: Human-Centered Development and Validation Study
Source: JMIR Hum Factors. 2026 Feb 23;13:e79952. doi: 10.2196/79952 (PMC12928693; doi:10.2196/79952)
Supplement: Multimedia Appendix 2 [file humanfactors-v13-e79952-s002.pdf]

Each record (a row in the below matrix) had a corresponding data visualization in the midpoint report. Each visualization in a live version would show a report owner's aggregate data for some element of their clinical experience or forecast potential future exposures (e.g., how many patients with a particular diagnosis had they seen in the last six months set against how many they might see on a particular elective). Our prototypes showed data from an "average trainee" conceived by study team members qualifying as subject matter experts in graduate medical education and clinical practice.

All visualizations were developed under the assumption that rPPI attribution can translate EHR metadata into a measure of trainee clinical exposure. The data for each visualization was described in the form of the structured query required to generate the visualization, adapted to more natural language.

All MV Midpoint Report visualizations are labeled as V1 in the below matrix. V2 and V3 records were deemed too challenging to build in an initial version given expected challenges to access and data engineering resources required.

Multimedia Appendix 2. Feature Prioritzation Matrix.

Record ID	Version	Feature / Visualization Title	Section (question answered by the graph)	What is literally displayed? (Analytic Logic in Pseudo SQL)	Date range for data pull	Impact Score (1 = low; 5 = high)
1.4	V1: MV Midpoint Report	Top CCSR Diagnosis Group Seen	My Last 6 Months	Count of diagnoses from user trainee's RPPI; Count of total patients that had an RPPI with user trainee. Group by CCSR diagnosis type. Show the top 10 diags by volume. Show count and show diag count as fraction of total patients.	Last 6 months	4

1.5	V1: MV Midpoint Report	Gaps in Diagnosis Exposure	My Last 6 Months	Count of diags from user trainee's RPPI. Count of diags for all same-year-as-user trainees. Count of diags from all trainees over past years (flexible number based on how hard this is to pull, target 3 years). Group the counts by diag and by whether they belong to the user trainee, their current cohort, or past years. Calc the difference between current cohort diag counts and user-trainee's diag count. Sort by the difference, descending. Show the top 10 diags by difference. Show counts for user trainee, cohort, and historic cohort where hist cohort divided by (total months of pull/6).	Last 6 months	4
2.1	V1: MV Midpoint Report	What diagnoses am I seeing Most?	Diagnosis Breakdown	Count of diags. Group by care setting (ED, ICUs, med wards, outpatient, and surgical) and by acuity level (when the care setting is the emergency department).	Last 6 months	4
7.1	V1: MV Midpoint Report	Top diagnoses by Δ of elective makeup vs my past patient makeup	Will I see important diagnoses (if I choose this elective)?	Count of all diags seen by same-year-as-user trainees. Group by diags. Show as a % of total. Show count of diags seen by the user-trainee as a comparison.	Last 6 months	4

3.7	V1: MV Midpoint Report	My Patient Makeup	What is my exposure to acuity and complex care?	Count of all user trainee's RPPI patients. Of those, fraction that had more than one readmission; fraction that had more than 3 emergency department visits in 12 months; fraction that had more than one chronic condition; fraction that had more than 5 medications; that suffer housing instability; that are active drug users.	Last 12 Months	3
1.6	V2: Future Version	Potentially Missed Exposure	My Last 6 Months	Count of all diags from RPPI with the user trainee. Sum of time user trainee opened charts of any patients seen by any clinician on the user trainee's shifts where. Where sum of time in those charts is <5minutes, treat that patient as a missed exposure. Count all diags from missed exposures. For all diags that makeup bottom 80% of actual exposure, show the top diags by missed exposure. Sum actual exposure to missed exposure counts as Potential Exposure. Group by diag. Show actual exposure by count and % of total exposure and show Potential exposure.	Last 6 months	4
6.2	V2: Future Version	What is the time commitment	What is the time commitment ?	time logged in the EHR from different terminals and devices: Mobile, Trainee Home machine, and CHOP machines	Last 6 months	4

7.2	V2: Future Version	Diagnoses my mentors want me to see (elective/clinic comparison)	Will I see important diagnoses?	Same as 7.1 but filtered on diags that fall into CCSR types that were selected by mentors as a target set for trainees to get experience with.	Last 6 months	4
9.3	V2: Future Version	Residents who took this elective	Who can I ask about it?	show other trainees who have taken the elective/clinic	Last 2 years	4
1.3	V2: Future Version	My Specializations	Overview (pre-graphs)	Short set of specialization interests (no more than 3) entered in by the user following a prompt when they first log into the system. Should be low character limit in the field entry form.	Last 6 months	3
6.3	V2: Future Version	Time Writing Notes	How active and demanding is it?	time spent writing any type of notes in an elective	Last 6 months	3
7.6	V2: Future Version	Continuity of Care (elective/clinic comparison)	How much exposure will I get to each patient?	number of RPPI average patients for same-year-as-user trainees at each level of a continuity of care metric (COCM). Two ideas for COCM metrics: [1] the validated Usual Provider of Care (UPC - see cited paper) metric [2] number of sequential visits to or encounters with each trainee. Group by COCM rounded to nearest whole number. Show data from graph 3.9 compared to same-year-as-user trainees data. doi: 10.1097/MLR.0000000000000018	Last 6 months	3
8.1	V2: Future Version	Rotation's Patient Makeup	Will I see complex care?	Same as 3.7 but for all same-year-as-user trainees.	Last 6 months	3

8.6	V2: Future Version	Concurrent EHR Sessions Per Day on the Same Patient	Will I coordinate care with other roles?	See reference articles for calculating metric. doi:10.2196/28998	Last 6 months	3
3.9	V2: Future Version	Continuity of Care	How much exposure am I getting to each patient?	Count of of RPPI patients for user trainee at each level of a continuity of care metric (COCM). Two ideas for COCM metrics: [1] the validated Usual Provider of Care (UPC - see cited paper) metric [2] number of sequential visits to or encounters with user trainee. Group by COCM rounded to nearest whole number. doi: 10.1097/MLR.0000000000000018	Last 6 months	2
3.1	V2: Future Version	New Patients	How much exposure am I getting to each patient?	Count of patients where patients had no prior history with the Unit or clinic and an RPPI with the user trainee is their first.	Last 6 months	2
5.2	V2: Future Version	Attention Switches	How efficient and acurate am I with the EHR?	See reference articles for calculating attention switches. https://doi.org/10.1016/j.bja.2022.04.012	Last 12 Months	1
2.2	V3: Distant Future Version	What diagnoses do my mentors want me to see?	Diagnosis Breakdown	Same as 2.1 but filtered on diags that fall into CCSR types that were selected by mentors as a target set for trainees to get experience with.	Last 6 months	4
1.1	V3: Distant Future Version	My Goals	Overview (pre-graphs)	Short set of goals (no more than 3) entered in by the user following a prompt when they first log into the system. Should be low character limit in the field entry form.	Last 6 months	3
1.2	V3: Distant Future Version	My Recent Electives	Overview (pre-graphs)	Pull list of electives in last 6 months	Last 6 months	3

3.1	V3: Distant Future Version	CAT Notes	What is my exposure to acuity and complex care?	Count of CAT notes written by the user trainee OR written about any RPPI patient of the user trainee's within +/- 24h that the user trainee saw them. Group by month.	Last 12 Months for raw data; filter on Hour +/- 24h of user trainee's shifts	3
3.2	V3: Distant Future Version	Watcher Notes	What is my exposure to acuity and complex care?	Count of Watcher notes written by the user trainee OR written about any RPPI patient of the user trainee's within +/- 24h that the user trainee saw them. Group by month.	Last 12 Months for raw data; filter on Hour +/- 24h of user trainee's shifts	3
3.5	V3: Distant Future Version	Any of the above	What is my exposure to acuity and complex care?	Sum patient volumes from 3.1 , 3.2, 3.3, and 3.4. Group by month.	Last 12 Months for raw data; filter on Hour +/- 24h of user trainee's shifts	3
3.6	V3: Distant Future Version	Time reading ICU consult notes	What is my exposure to acuity and complex care?	Sum of user trainee time spent reading notes where notes are written by ICU consults.	Last 6 months	3

4.7	V3: Distant Future Version	Sample Quality by BX Type x Imaging	What is my exposure to procedures?	Average of sample quality scores from Procedure Master (/Model?) File for all biopsies where user trainee performed the biopsy. Compare to same but performed by attengings only during user trainee's shifts. Should show if the samples fellows are getting are actually good samples? Are the quality scores of the samples from radiology improving?	Last 6 months	3
5.1	V3: Distant Future Version	Time reading consult notes by dept	Do I see coordinated care?	Sum of time for all same-year-as-user trainees spent reading notes where notes are written by consults. Group by consult's department. Get average time/week for user trainee.	Last 6 months	3
7.3	V3: Distant Future Version	Acute Patient Volume	Will I see acuity?	Count of all patients with acute criteria (CAT notes, Watcher notes, ICU Transfers, or patients put on ventilator or pressors) in the elective. Get weekly average. Compare to sum from graph 3.5 made into weekly average.	Last 6 months	3
7.4	V3: Distant Future Version	Time Reading Consult Notes (elective/clinic comparison)	Will I see acuity?	Sum of time for all same-year-as-user trainees spent reading notes where notes are written by consults. Group by consult's care setting (ICU, surgical ward, medical ward). Get average time/week for rest of class vs trainee.	Last 6 months	3

7.5	V3: Distant Future Version	Patients By Phase In Their Hospital Course	How much exposure will I get to each patient?	Patient volume where same-year-as-trainees were the provider during any of the following buckets: (1) Admission ONLY (2) Admission AND any other care before discharge (3) any care between discharge AND NOT Admission AND NOT Discharge (4) any care before discharge AND discharge (5) Discharge ONLY (6) Admission AND any care AND discharge. Compare to user trainee's stats for each bucket from 3.8	Last 6 months	3
8.7	V3: Distant Future Version	Time reading consult notes by dept (elective/clinic comparison)	Will I coordinate care with other roles?	Same as 5.1 but instead, pull for all same-year-as-user trainees in the elective/clinic.	Last 6 months	3
3.3	V3: Distant Future Version	Transferred to ICU	What is my exposure to acuity and complex care?	Count of transfers to ICU of any RPPI patient of the user trainee's within +/- 24h that the user trainee saw them. Group by month.	Last 12 Months for raw data; filter on Hour +/- 24h of user trainee's shifts	2

3.8	V3: Distant Future Version	Hospital Course Exposure	How much exposure am I getting to each patient?	Count of patients where user Trainee was the provider during any of the following buckets: (1) Admission ONLY (2) Admission AND any other care before discharge (3) any care between discharge AND NOT Admission AND NOT Discharge (4) any care before discharge AND discharge (5) Discharge ONLY (6) Admission AND any care AND discharge	Last 6 months	2
4.1	V3: Distant Future Version	Procedures Performed by Month	What is my exposure to procedures?	Count of procedures where user trainee led or assisted the procedure or where an attending performed the procedure when the trainne was on shift. Group by user trainee involvement vs not (shown as attending's procedure) and by month.	Last 12 Months	2
4.2	V3: Distant Future Version	By Care Setting	What is my exposure to procedures?	Same as 4.1 but instead, second Group By is care setting (ICU/Surgical/Medical)	Last 6 months	2
4.3	V3: Distant Future Version	By Procedure Type	What is my exposure to procedures?	Same as 4.1 but instead, second Group By is CCSR procedure type (major/minor x theraputic/diagnostic). Map from ICD 10 codes here: https://www.hcup-us.ahrq.gov/toolssoftware/ccsr/prccsr.jsp	Last 6 months	2
4.4	V3: Distant Future Version	By Procedure	What is my exposure to procedures?	Same as 4.1 but instead, second Group By is is the procedure itself	Last 6 months	2

4.5	V3: Distant Future Version	Education by Method and Response to it	Is my patient education skillset improving?	Count of "Method" and "Response" values in the Epic Education Topic data entry form. Group by method, then by response. Response should be shown as a percent of all responses in that particular method. For example, when the method is "Handout" show the percent of all Handout encounters for each of the five response values.	Last 6 months	2
4.6	V3: Distant Future Version	Missed opportunities to repeat existing education	Is my patient education skillset improving?	Count of RPPI where either of the two preceding encounters (regardless of the clinician involved) included new education AND the user trainee (during their RPPI) did not repeat/reinforce the topic.	Last 12 Months	2
6.4	V3: Distant Future Version	Watcher Notes Written	How active and demanding is it?	time spent writing ONLY watcher notes	Last 6 months	2
6.5	V3: Distant Future Version	Phone Calls to Families	How active and demanding is it?	Number of phone call notes or calls logged otherwise in EHR	Last 6 months	2

8.2	V3: Distant Future Version	What procedures will I see?	What procedures will I see?	Count of procedures where same-year-as-trainee population performed the procedures and procedures where up-authority personnel performed the procedure and not the trainee. For a resident trainee, also count procedures performed by fellows and attendings. For a fellow trainee, also count the attending procedures. Group by trainee performed procedures, by up-authority performed procedures, and by procedure itself.	Last 6 months	2
8.3	V3: Distant Future Version	Education Method Volume	Will my education skillset grow?	Similar raw data to 4.5 Count of encounters for all same-year-as-user trainees where education topic data was entered. Group by education method. Compare to count of encounters for the same thing for User trainee. Show average by week.	Last 6 months	2
8.4	V3: Distant Future Version	Education Response	Will my education skillset grow?	Similar raw data to 4.5 Count of encounters for all same-year-as-user trainees where education topic data was entered. Group by response to education. Compare to count of encounters for the same thing for User trainee. Show average by week.	Last 6 months	2

8.5	V3: Distant Future Version	Education Repetition Opportunities	Will my education skillset grow?	Similar to 4.6<br>Count of RPPI in the elective/clinic where either of the two preceding encounters (regardless of the clinician involved) included new education (implying that counted encounter got the chance to repeat/reinforce the topic). Compare to same value for user trainee.	Last 6 months	2
9.1	V3: Distant Future Version	Total Labs and Imaging Ordered	Will it be a diagnostic stewardship and interpretation challaenge?	For all same-year-as-user trainees, number of weekly labs and images ordered in the elective/clinic. Compare to user trainee's max and minimum orders of the same diagnostics for all weeks.	Last 6 months	2
9.2	V3: Distant Future Version	Top 10 Diagnostic Orders	Will it be a diagnostic stewardship and interpretation challaenge?	Same as 9.1 but grouped by Diagnostic.	Last 6 months	2
3.4	V3: Distant Future Version	Put on Vent or Pressors	What is my exposure to acuity and complex care?	Count of user trainee's RPPI patients who were put on a ventilator or vasopressors within +/- 24h that the user trainee saw them. Group by month.	Last 12 Months for raw data; filter on Hour +/- 24h of user trainee's shifts	1
5.3	V3: Distant Future Version	Medication Errors	How efficient and acurate am I with the EHR?	See reference articles for calculating metric. DOI: 10.1097/PTS.0000000000001011	Last 12 Months	1
5.4	V3: Distant Future Version	Wrong-Patient Errors	How efficient and acurate am I with the EHR?	See reference articles for calculating metric https://doi.org/10.1016/j.bja.2022.04.012.	Last 12 Months	1

5.5	V3: Distant Future Version	Ambulatory EHR Efficiency	How efficient and acurate am I with the EHR?	See reference articles for calculating metric. DOI: 10.1055/s-0041-1732403	Last 12 Months	1
5.6	V3: Distant Future Version	Time writing messages that are opened for >1 second or not at all	How efficient and acurate am I with the EHR?	See reference articles for calculating metric https://www.aspe.hhs.gov/sites/default/files/migrated_legacy_files/194056/jsk-qebhr-final-concept-report.pdf.	Last 12 Months	1
