# Supplementary material for: A Log-Level Data-Driven Precision Education Tool for Pediatrics Trainees: Human-Centered Development and Validation Study
Source: JMIR Hum Factors. 2026 Feb 23;13:e79952. doi: 10.2196/79952 (PMC12928693; doi:10.2196/79952)
Supplement: Multimedia Appendix 5 [file humanfactors-v13-e79952-s005.pdf]

Multimedia Appendix 5. Quotes and Their Throughline To Study Phase Outputs

Results Data: Throughline of All Phases to Task 1			
**Phase 1 Work Domain Assessment: Quote & Coded Topics from Provisional Coding**		**Phase 2 Formative Usability Testing: Relevant Visualisation from our Education Decision Support Tool (MV Midpoint Report)**	**Phase 3 Summative Usability Testing: Task Using That Visualization**
"I don't think you feel comfortable until you've managed [a diagnosis] on your own. So, I think repetition is one [path to comfort], and then having that repetition with autonomy. When you're the one making the decisions and the responsibility is on you."	People; Tasks	Top diagnoses I have seen, total aggregate	Scenario: You are on inpatient servcice with three patients, problem lists given for all three.<br><br>Question: Pick the patient for whom you should ask for more autonomy.<br><br>[One problem list has frequently seen dx by the participant. The other two do not]
"I don't think that there really exists too much of a… good mechanism to track what I've seen, outside of qualitatively recording in my head that 'oh yeah, I feel pretty comfortable with asthma. I feel like I've seen a number of teenage asthma kids in different levels of disease, and maybe I've seen a couple less, compared to some of my colleagues, of reactive airway disease in younger kids, and especially sicker one, but have seen a bronchiolitis' "	People; Tools; Tasks		
"you probably know the conditions of the kids that you've frontlined for…the learning and comfort level comes at times when you're challenged to take responsibility"	People; Tasks		

Results Data: Throughline of All Phases to Task 2			
**Phase 1 Work Domain Assessment: Quote & Coded Topics from Provisional Coding**		**Phase 2 Formative Usability Testing: Relevant Visualisation from our Education Decision Support Tool (MV Midpoint Report)**	**Phase 3 Summative Usability Testing: Task Using That Visualization**

<table>
<tr><td>"So, there’s just the background knowledge, so either a didactic session or an article on UpToDate, or even primary literature, that’s one bucket of “I know what to do,” like, or I’ve read about what I should do in this situation. A good personal example is I’m on adolescent right now. I haven’t treated a single STI, even to date. But I’ve read about it. And we have had didactics on it, and we’ve had primary literature on it. And so I have a good sense of what I should be doing. That’s part one. At least I have the background knowledge."</td><td>People; Tools; Tasks</td><td rowspan="3">Gaps in my diagnosis exposure against cohort average</td><td rowspan="3">Scenario: In an upcoming journal club or EBP presentation, you must pick the topic.

Question: Choose a disease for an article or case in which you lack exposure while your peers may bring in deep experience.

[Several diagnoses from the graph fit this criteria]</td></tr>
<tr><td>"I use UpToDate. As I've gotten further into my residency, I've used PubMed and primary literature reviews more. I'll say I have not gone cowboy and read something and just do it in the moment. But where treatment course needs to go for some of the more rare stuff is helpful for me. Iit allows me to make simple decisions without having to ask someone constantly"</td><td>People; Tools; Tasks</td></tr>
<tr><td>"[on exposure that leads to mastery] Sometimes, people will say, 'well, there were six asthmatics on my team. I wasn’t primarily responsible – like, the primary clinician for them, but I discovered them, or like I participated in discussions on rounds about them.' That feels like I’ve had exposure"</td><td>People; Environment; Tasks</td></tr>
</table>

<table>
<tr><th colspan="3">Results Data: Throughline of All Phases to Task 3</th></tr>
<tr><th>Phase 1 Work Domain Assessment: Quote & Coded Topics from Provisional Coding</th><th>Phase 2 Formative Usability Testing: Relevant Visualisation from our Education Decision Support Tool (MV Midpoint Report)</th><th>Phase 3 Summative Usability Testing: Task Using That Visualization</th></tr>
</table>

<table>
<tr><td>"I think it could play in terms of the emergency department, to say 'oh, actually I haven't taken care of many of these sick kids,' so if there were an option to pick between a couple,... that would still be helpful, thinking and going into and making learning goals...to have [aggregated practice data] to back that up"</td><td>Environment; Tools; Tasks</td><td rowspan="2">By care setting (and by acuity where recorded), top diagnoses I have seen</td><td rowspan="2">Scenario: You are in a critical care setting with three patients, problem lists given for all three.

Question: Pick the patient for whom you might want more help or supervision.

[All patient problem lists have items from participant's top dx. For only one of the three patients, participant has no experience with their dx in critical care]</td></tr>
<tr><td>"[asked about what produced self-efficacy] being in the PICU and the NICU, you get those basics… We see weird stuff here all the time, that you're exposed to just once or twice. I usually read the moment, but I think a lot of it is just having some sort of backbone of how to like generally care for a sick child.."</td><td>Environment; People; Tasks</td></tr>
</table>

<table>
<tr><th colspan="4">Results Data: Throughline of All Phases to Task 4</th></tr>
<tr><th colspan="2">Phase 1 Work Domain Assessment: Quote & Coded Topics from Provisional Coding</th><th>Phase 2 Formative Usability Testing: Relevant Visualisation from our Education Decision Support Tool (MV Midpoint Report)</th><th>Phase 3 Summative Usability Testing: Task Using That Visualization</th></tr>
<tr><td>"But, if [your senior residents] are like " You want to treat with that? It wouldn't be my decision, but we'll do it." And then you own it. You have more ownership and comfort. The problem is some people may not feel comfortable until they see 50 [cases], and some people may not feel comfortable until they see 100. Everyone's learning curve is different. It's volume, complexity, and autonomy [driving learning], because obviously the more complex the issue you're managing on top of the patient comorbidities, then with the autonomy, and then how many you've managed. So, if you’ve managed an asthmatic, you feel comfortable, but then you have an asthmatic with CF? That adds a different layer."</td><td>People; Tasks</td><td>My exposure by patient count to elements of complex care</td><td>Scenario: You are on service with three patients assigned to you, problem lists given for all three.

Question: Pick the patient for whom you should ask for more autonomy.

[One of the patients is clearly a complex care patient of the type that participant has extensive experience]</td></tr>
</table>

Results Data: Throughline of All Phases to Task 5

Phase 1 Work Domain Assessment: Quote & Coded Topics from Provisional Coding		Phase 2 Formative Usability Testing: Relevant Visualisation from our Education Decision Support Tool (MV Midpoint Report)	Phase 3 Summative Usability Testing: Task Using That Visualization
"Having that [aggregate practice] data beforehand, and even potentially reviewing it with your mentor could be helpful. And even reviewing it every year. That would be helpful, a check-in. That could be the end-of-the-year check-in. Like, this is what you've done this year. That might pair nicely going back to the ILP, like these are my goals. This is what you've seen. Did they match up? Was that what you were trying to do with your goals? And what are your goals for next year? You can log it in your next ILP. Going forward beyond choosing rotations, choosing the types of patients in settings where you're allowed to choose patients could be helpful."	People; Environment; Tools; Tasks	By elective, gaps in my diagnosis exposure against those seen on the elective	Scenario: You have three available electives to choose from.<br><br>Question: Which will expose you to diagosis and management of diseases least familiar to you?<br><br>[One of the electives has far greater diagnosis Δs against participant's past exposure than the others]
"It'd be helpful second and third year, with elective time on a certain career track, to say 'hey, looking at this, I really don't have many ambulatory experiences. I really have seen very little sports med kids.' "	People; Tools; Tasks		
"I'll do a self-check in – going into third year when we start designing our schedules, of what have I not really seen a lot of? What would I like to see more of? That may help me figure out – maybe I'll do a sub-specialty IU just to get more of a certain type of diagnosis or patient, or I might gear it towards an acuity standpoint, more of the one triple of six electives that gets you more exposure with critical care."	Environment; People; Tools; Tasks		
