# Supplementary material for: A Log-Level Data-Driven Precision Education Tool for Pediatrics Trainees: Human-Centered Development and Validation Study
Source: JMIR Hum Factors. 2026 Feb 23;13:e79952. doi: 10.2196/79952 (PMC12928693; doi:10.2196/79952)

# Process Map - Resident - Elective Picking

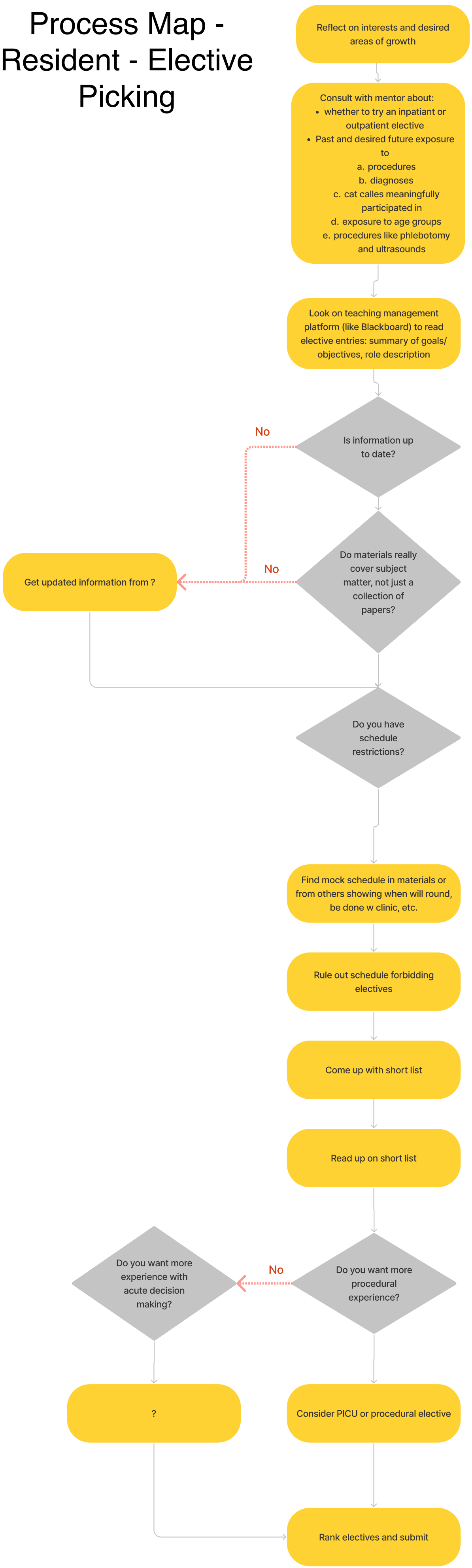

# Process Map - Mentor - Residency

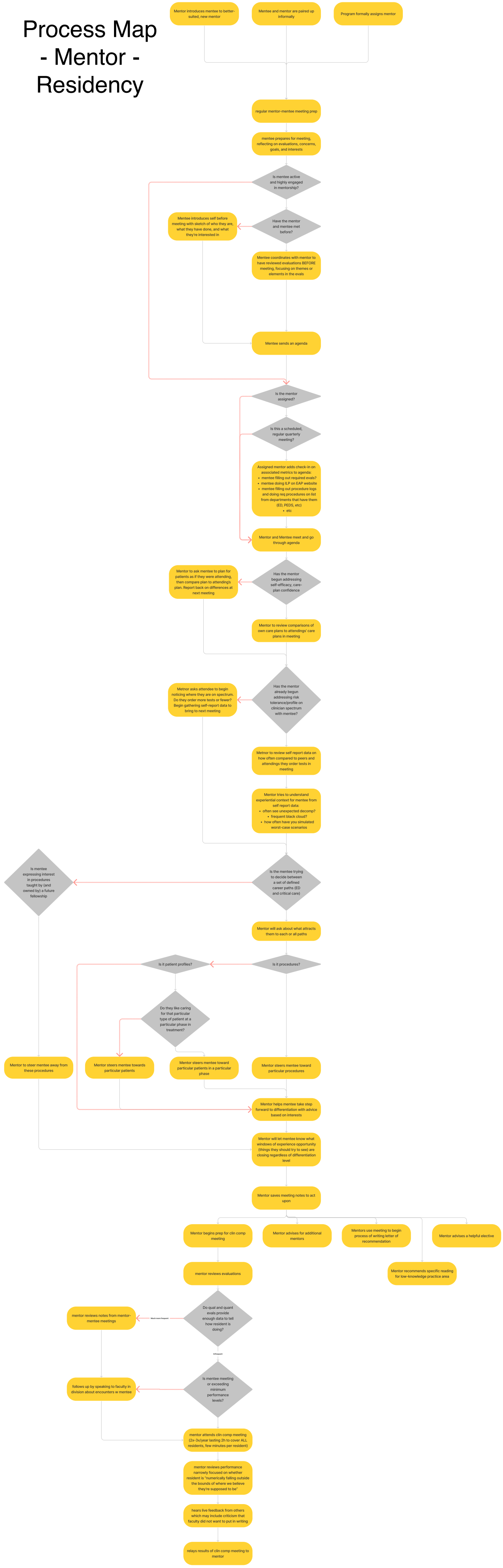

# Process Map - Resident - Mid Rotation

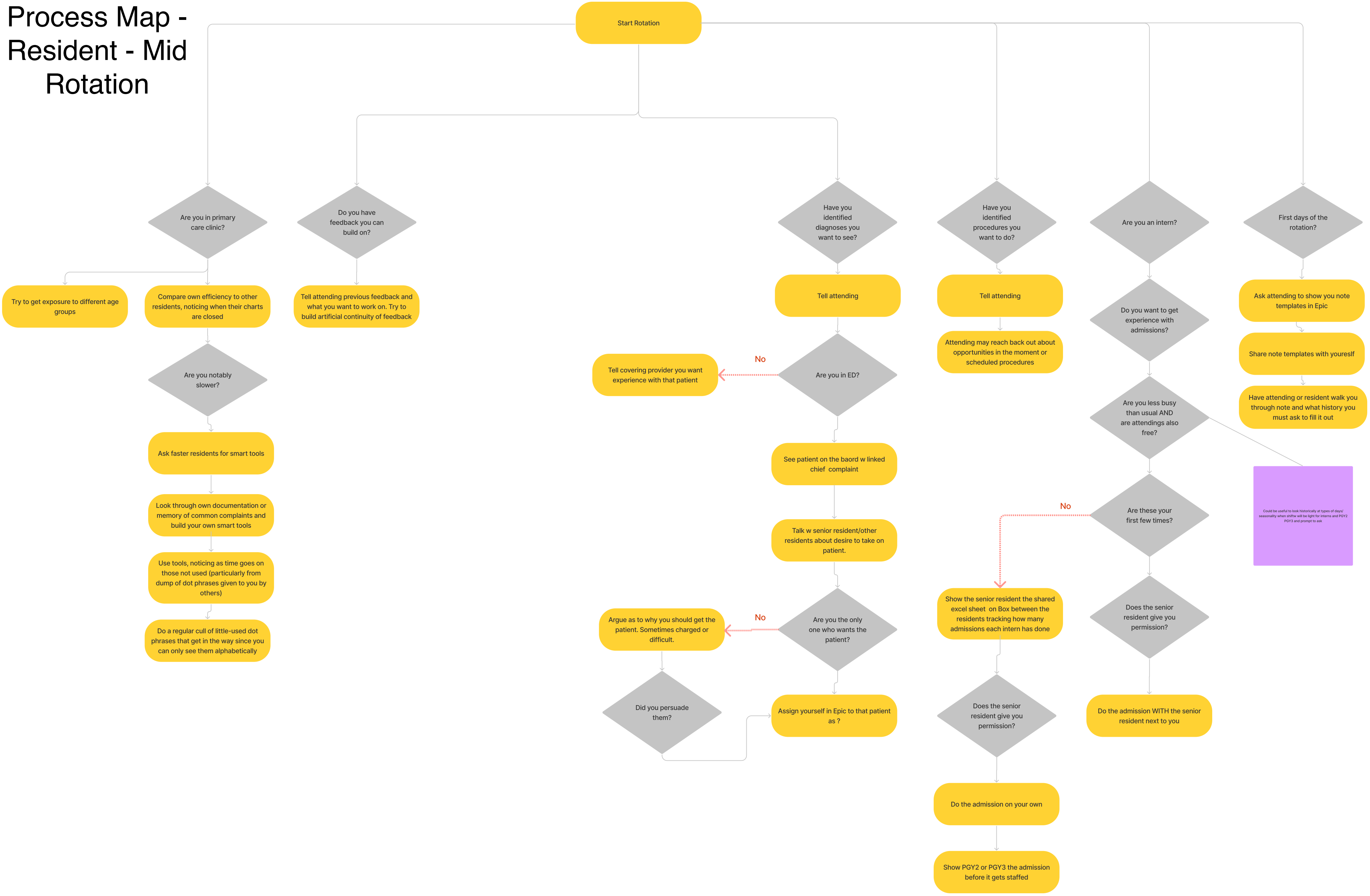

# Process Map - Resident - Pre Rotation

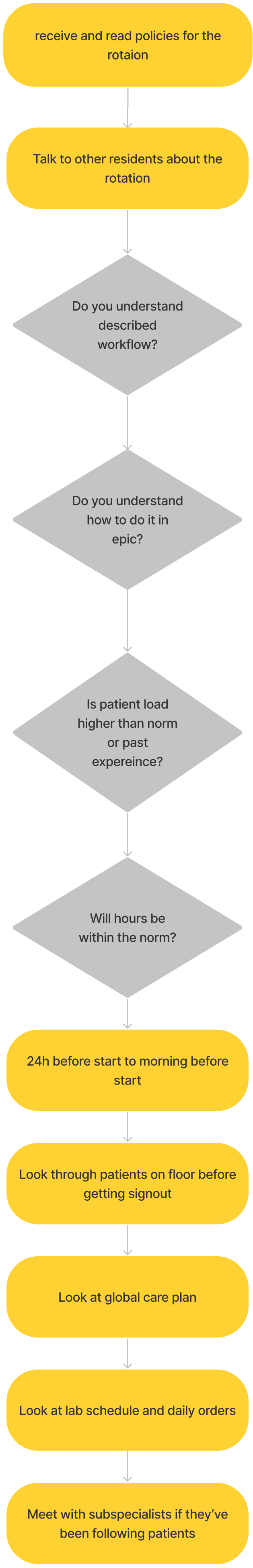

# Process Map - Program Director - Evaluations

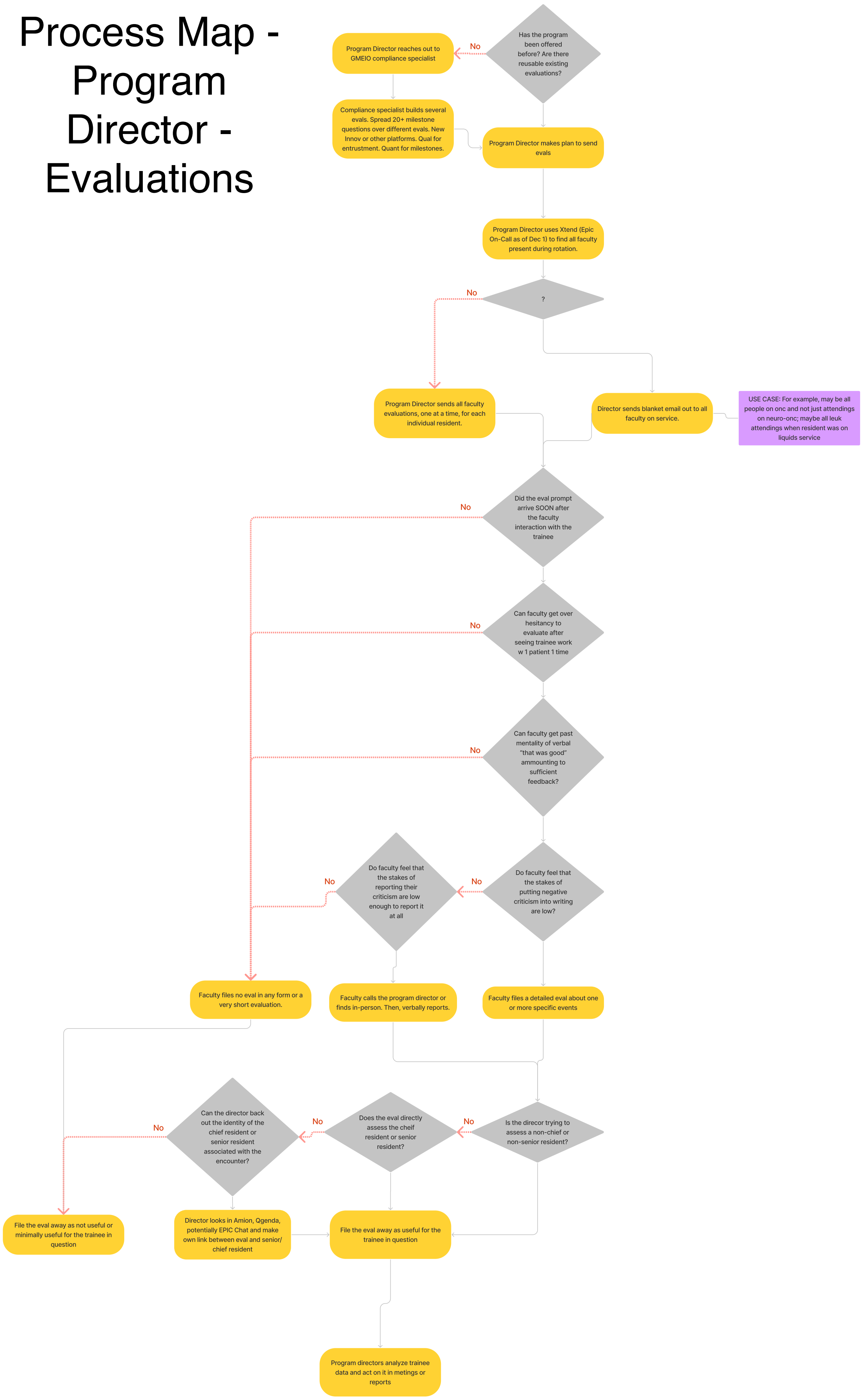

# Process Map - Program Director - New Policy Creation

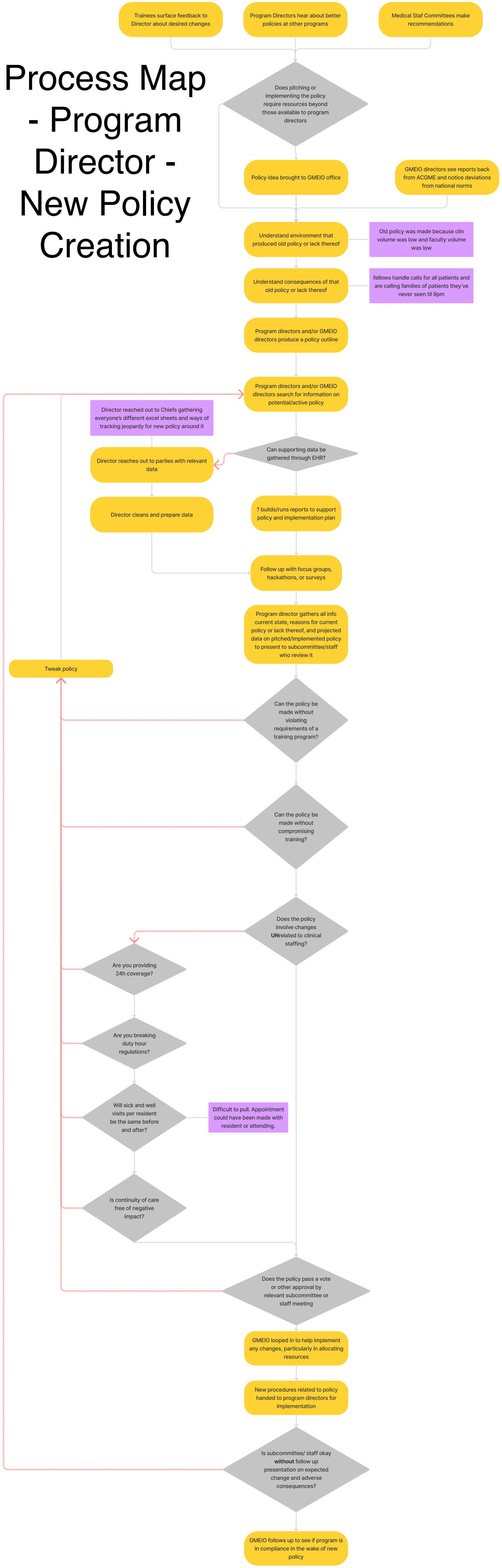

# Process Map - Program Director - Flow of Information to ACGME

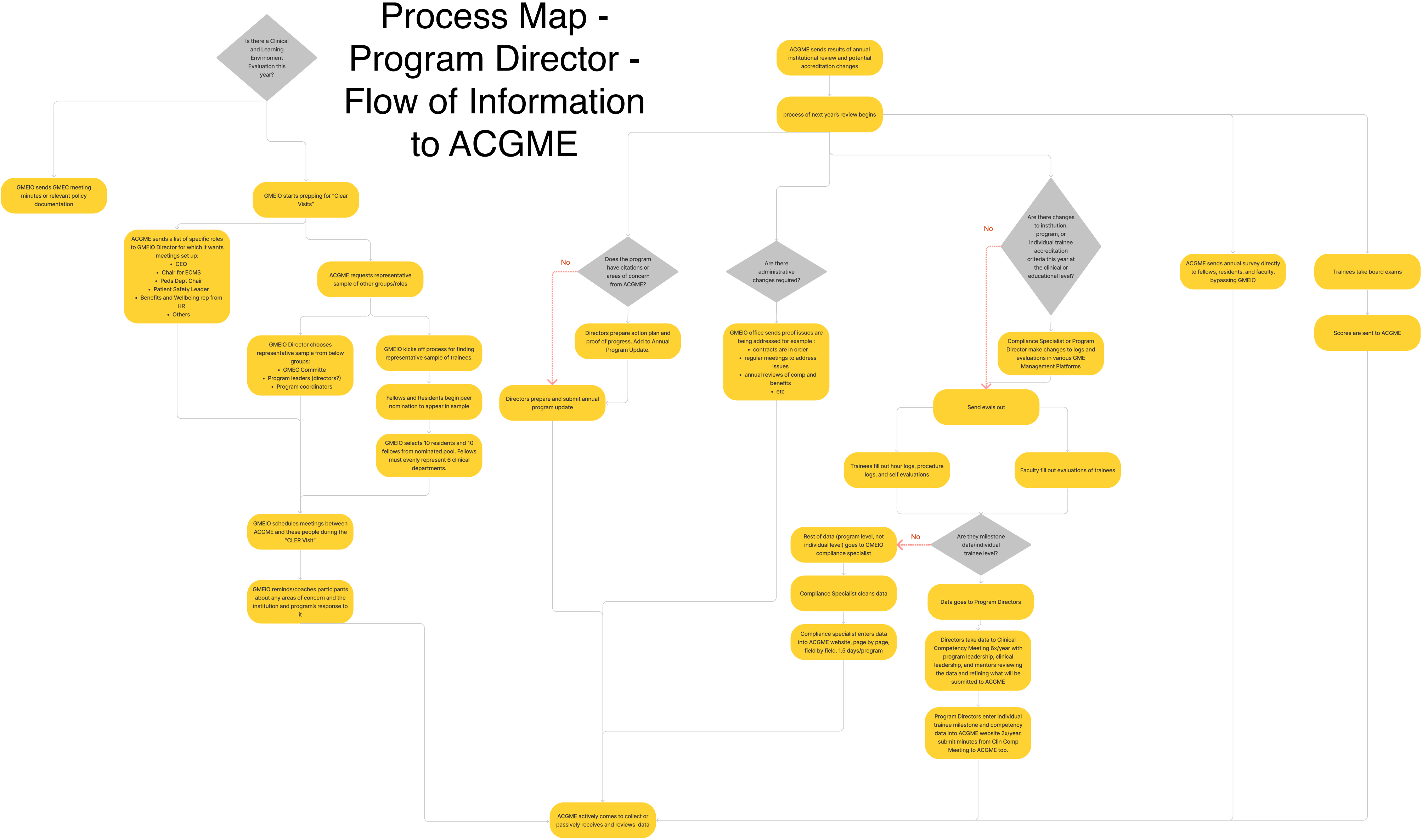

Supplement: Multimedia Appendix 6 [file humanfactors-v13-e79952-s006.pdf]
